# Calculation of the Contact Angle of Polymer Melts on Tool Surfaces from Viscosity Parameters

**DOI:** 10.3390/polym10010038

**Published:** 2017-12-30

**Authors:** Gernot Zitzenbacher, Hannes Dirnberger, Manuel Längauer, Clemens Holzer

**Affiliations:** 1 Department of Materials Technology, School of Engineering, University of Applied Sciences Upper Austria, 4600 Wels, Austria; hannes.dirnberger@fh-wels.at (H.D.); manuel.laengauer@fh-wels.at (M.L.); 2 Department Polymer Engineering and Science, Chair of Polymer Processing, Montanuniversitaet Leoben, 8700 Leoben, Austria; clemens.holzer@unileoben.ac.at

**Keywords:** polypropylene, polymethylmethacrylate, contact angle, viscosity, tool surface, wetting, injection molding, extrusion

## Abstract

It is of great importance for polymer processing whether and how viscosity influences the wettability of tool surfaces. We demonstrate the existence of a distinct relationship between the contact angle of molten polymers and zero shear viscosity in this paper. The contact angle of molten polypropylene and polymethylmethacrylate on polished steel was studied in a high temperature chamber using the sessile drop method. A high pressure capillary rheometer with a slit die was employed to determine the shear viscosity curves in a low shear rate range. A linear relation between the contact angle and zero shear viscosity was obtained. Furthermore, the contact angle and the zero shear viscosity values of the different polymers were combined to one function. It is revealed that, for the wetting of tool surfaces by molten polymers, a lower viscosity is advantageous. Furthermore, a model based on the temperature shift concept is proposed which allows the calculation of the contact angle of molten polymers on steel for different temperatures directly from shear viscosity data.

## 1. Introduction

Many phenomena in polymer processing are influenced by the wettability of solid surfaces by polymer melts. The wettability of solid surfaces in tools and molds affects the replication of surface structures and remolding forces in injection molding. As an example, in micro injection molding the wettability of the mold surface and the temperature dependence of viscosity near the glass transition temperature are important for the replication of surface structures [1]. Ejection forces decrease linearly with the contact angle of the polymer melt on the mold coating [2]. Rytka et al. [3] reported that the dewetting potential correlates well with the replicated height of different mold structures. A lower dewetting potential of a polymer leads to a better replication accuracy.

Wettability also affects the polymer melt flow in flow channels and dies. Fluoropolymers on die surfaces influence wall slip and shark skin in polymer melt rheology. Hatzikiriakos et al. [4] investigated the effect of fluoropolymer coatings on high density polyethylene (HDPE). Seidel et al. [5] found that polyetheretherketone (PEEK) and polytetrafluoroethylene (PTFE) induce wall slip of the polymer melt and cause a smooth extrudate surface. Agassant et al. [6] reported that coatings such as PTFE, which enhance slip at the wall, reduce or even eliminate sharkskin.

Hard coatings, which are often applied to screws and tools by physical vapor deposition (PVD) or plasma assisted chemical vapor deposition (PACVD), and metals can also influence the polymer melt flow and the extrudate appearance. Ramamurthy [7] studied the effect of different metals on wall slip and on the extrudate appearance of polyethylene. He observed that beryllium copper as a die material causes a lower critical wall shear stress for the onset of wall slip compared to steel. Rauwendaal [8] reported that the reduction of the pressure loss in a spiral mandrel extrusion die with a Lunac coating can be obtained. Zitzenbacher et al. [9,10] investigated the effect of diamond-like carbon (DLC), titanium nitride (TiN), titanium aluminum nitride (TiAlN), and steel on wall slip of a polypropylene copolymer and polymethylmethacrylate (PMMA). It was observed that a higher polarity as exhibited by TiN reduces wall slip of PP, especially at higher temperatures. PMMA slips only on polished steel, but no slip was found on DLC.

Young’s equation [11] is needed to describe the wetting of a solid surface by a liquid

(1) $$\sigma_{SL}=\sigma_{S}-\sigma_{L}cos\theta$$

It is obtained from a balance of the surface energy between the liquid and the solid σ_SL_, the surface energy of the solid σ_S_ and the surface tension of the liquid σ_L_ including the contact angle θ at the three phase points of liquid, solid, and surrounding atmosphere. According to Kumar [12] a complete wetting (θ = 0°), a partial wetting (0°< θ < 90°), a partial non-wetting (90° < θ < 180°) and a total non-wetting (θ = 180°) can be distinguished.

Although the wettability of solid substrates is often determined at room temperature, the contact angle in the polymer melt state is needed for the explanation of phenomena which are related to the interface between polymers and tool or screw materials. Schonhorn et al. [13] investigated the wetting of aluminum, mica, and Teflon by an ethylene-vinyl acetate copolymer and polyethylene melts. Silberzan et al. [14] studied the spreading behavior of poly(dimethylsiloxane) (PDMS) liquids on silica surfaces at room temperature through optical microscopy and ellipsometry. Anastasiadis et al. [15] investigated the wetting of molten linear low density and high density polyethylene on steel and fluoropolymer coatings. Wulf et al. [16] studied the surface tension of a polystyrene melt dependent on temperature in a sessile drop experiment. Wouters et al. [17] determined the surface tension of epoxy resins, polyesters, and additives. Kopczynska [18] and Yang et al. [19] investigated the surface tension of molten polycarbonate (PC), polystyrene (PS), styrene acrylonitrile (SAN), polyethylene (PE), and polyamide 6 (PA 6). Zitzenbacher et al. [20] studied the contact angle of molten polypropylene (PP), HDPE, PMMA, and Polyamid 6.6 (PA 6.6) on steel and different coatings such as titanium aluminum nitride (TiAlN), titanium nitride (TiN), chromium nitride (CrN), silicone doped diamond-like carbon (DLC), and polytetrafluoroethylene (PTFE). They observed a decrease in the contact angle of the molten polymers with a rising surface energy of the coating. Vera et al. [21] investigated the contact angle of molten PP, acrylonitrile butadiene styrene (ABS), and PC on steel, different titanium nitride coatings (TiNO*_x_*, TiNO*_y_*, and TiNO*_z_*), CrN, and DLC. Furthermore, they measured the surface tension of the molten polymers and evaluated work of adhesion on the solid substrates.

It is of great importance for polymer melt flow and surface structure replication whether and how viscosity influences the wettability of tool surfaces, especially in processes such as injection molding and extrusion technology. Furthermore, shear viscosity curves are often more easily available, but the contact angle of polymer melts on solid surfaces can only be determined in time consuming experiments. The goal of this paper was to study the influence of shear viscosity on the contact angle of molten polymers on tool steel. The contact angle was determined employing the sessile drop method in a high temperature chamber and the shear viscosity curves were measured in a low shear rate range using a high pressure capillary rheometer with a slit die. Furthermore, the contact angle and the shear viscosity curves were determined at elevated temperatures. It is demonstrated that the contact angle of the molten polymers is a function of zero shear viscosity. The data of the two different polymeric materials, PP and PMMA, are combined to one function. As a final result, a model is proposed to calculate the contact angle of polymer melts on steel for different temperatures directly from zero shear viscosity.

## 2. Materials and Methods 

A polypropylene homopolymer (PP) HD 120 MO, produced by Borealis, Linz, Austria, and a polymethylmethacrylate (PMMA) PLEXIGLAS 7M from Evonik, Darmstadt, Germany, were investigated in this study in order to gain a better understanding about their processing behavior. PP HD 120 MO has a melt flow rate (MFR) of 8 g (10 min)^−1^ at 230 C/2.16 kg and is used for injection molding applications. PMMA PLEXIGLAS 7M has a MFR of 3.45 g (10 min)^−1^ at 230 °C/3.80 kg and is employed for extruded profiles and panels. The MFR values mentioned were taken from the material datasheets. The testing temperature range of the polymeric materials was chosen according to the material suppliers’ recommended melt temperature range. PP was studied at a temperature of 185, 200, 210, and 220 °C. The testing temperature of PMMA was 230, 240, 250, and 260 °C, respectively. PMMA was predried before the experiments.

The viscosity curves of these polymeric materials were determined using a high pressure capillary rheometer Rheograph 6000, produced by Goettfert, Buchen, Germany, with a slit die. The slit die concept was employed because it allows the measurement of viscosity data in a low shear rate range, which was chosen between 0.1 and 350 s^−1^. Furthermore, directly measured pressure data is obtained which leads to true wall shear stresses, without having to use Bagley correction. First, the polymeric material was filled into the reservoir channel, melted, and heated up to the defined temperature. The slit die was heated to the same temperature. When extruding the melt, the piston velocity was increased stepwise in order to obtain rheological data at different apparent wall shear rate values. The wall shear stress τ_w_ was evaluated according to Walters [22]

(2) $$\tau_{w}=\frac{HW}{2\left(H+W\right)}\frac{\Delta p}{\Delta L}$$

where *H* is the height of the flow channel, *W* is the width of the flow channel, Δ*L* is the distance between the pressure transducers, and Δ*p* is the measured pressure loss.

The apparent wall shear stress is

(3) $${\dot{\gamma }}_{ap\;}=\;\frac{6Q}{WH^{2}}$$

where *Q* is the volume flow rate.

The Weissenberg–Rabinowitsch equation [23,24] was used to obtain the true shear rate at the wall

(4) $${\dot{\gamma }}_{w}=\;\frac{{\dot{\gamma }}_{ap}}{4}\left[3+\frac{d\left(log{\dot{\gamma }}_{ap}\right)}{d\left(log\tau_{w}\right)}\right]$$

Shear viscosity η can then be evaluated by introducing Equations (2) and (4) in Equation (5)

(5) $$\eta =\frac{\tau_{w}}{{\dot{\gamma }}_{w}}$$

The measurement and evaluation of the viscosity dependent on shear rate was conducted at least four times at each temperature. A mean value was calculated from these data at each shear rate value to obtain the final viscosity curve. The shear viscosity curve was approximated with the Bird-Carreau-Yasuda model [24] using the least squares method

(6) $$\eta \left(\dot{\gamma }\right)=\eta_{0}{\left[1+{\left(\lambda \dot{\gamma }\right)}^{a}\right]}^{\frac{n-1}{a}}$$

where η_0_ is zero shear viscosity, λ is a time constant, *n* is the Power Law index, and a accounts for the width of the transition region between zero shear viscosity and the Power Law region.

A Drop Shape Analyzer DSA 30S, produced by Kruess, Hamburg, Germany, with a high temperature chamber TC 21 was employed to measure the contact angle of the molten PP and PMMA on steel with the sessile drop method. The experiments were carried out under nitrogen atmosphere with a gas flow rate of 20 NL·h^−1^ to avoid thermooxidative degradation of the polymeric materials. A schematic diagram of the test setup is shown in [20].

The X38CrMoV5 1 steel discs with a diameter of 40 mm and a thickness of 10 mm were heat treated and polished to an area-weighted surface roughness *S*_a_ of 15.1 ± 5.7 nm. The surface roughness of the steel samples was determined by means of a confocal microscope DCM3D, Leica Microsystems, Wetzlar, Germany at three positions on the sample, one in the center, and two 10 mm from the center, once in *x*- and once in *y*-direction. Before the contact angle measurements the steel discs were cleaned carefully with isopropanol using a tissue and dried with an air stream. Afterwards, the steel sample was placed in the high temperature chamber and preheated 15 min to obtain a uniform temperature distribution.

In the next step, the polymer sample was placed on the solid surface with a pair of tweezers. The positioning of the polymer sample was conducted as fast as possible to avoid substantial temperature decrease and high nitrogen loss in the high temperature chamber. After melting the polymer sample, the drop shape was recorded dependent on time with a CCD camera with a frame rate of 1 fps. The contact angle was evaluated using the recorded video data. The drop contour was approximated with a polynomial function near the base line. The slope of the approximation function in the contact point of the three phases was used to evaluate the contact angle. Each test was carried out at least three times in order to verify its reproducibility and a mean value was calculated.

## 3. Results

### 3.1. Shear Viscosity Curves

**Figure 1:**
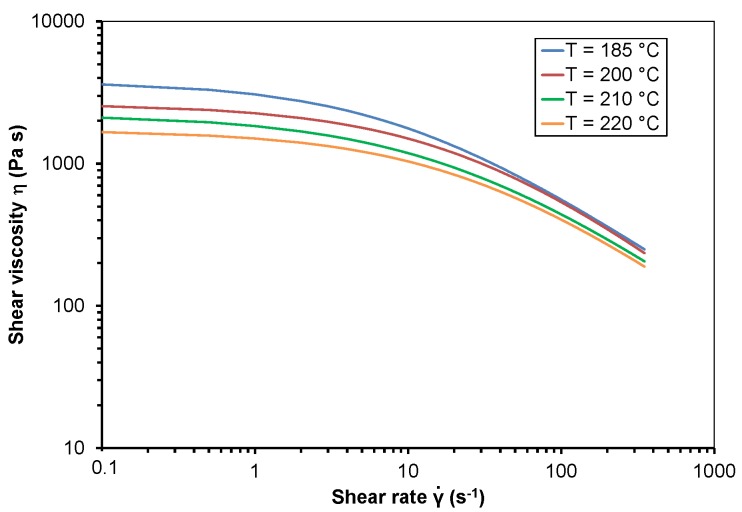
Shear viscosity of polypropylene dependent on shear rate at different temperatures.

**Figure 2:**
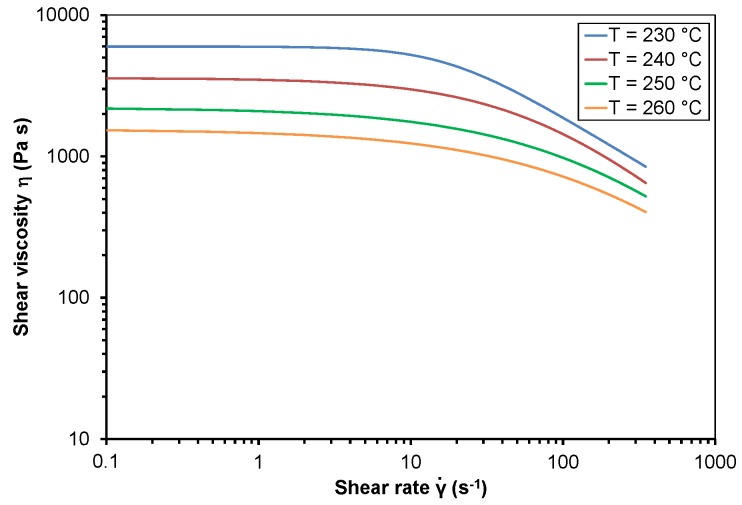
Shear viscosity of polymethylmethacrylate dependent on shear rate at different temperatures.

The shear viscosity of PP and PMMA dependent on shear rate at different temperatures is represented as the approximated curves in Figure 1 and Figure 2. Table 1 shows the Bird-Carreau-Yasuda parameters at different temperatures *T*, including the zero shear viscosity η_0_, the time constant λ, the Power Law index *n*, the width of the transition region a and the coefficient of determination *R*². The good reproducibility of the tests is indicated by the standard deviation of the measured shear viscosity values which is dependent on shear rate and temperature between 0.5% and 4.8%.

Both polymer melts exhibit shear thinning behavior, which means that shear viscosity decreases with rising shear rate. Furthermore, shear viscosity decreases with rising temperature. The shear viscosity curve of PP reveals a negative slope even at low shear rates. The width of the Newtonian tableau of PMMA decreases with rising temperature. The good approximation of the measured viscosity data with the Bird-Carreau-Yasuda model is indicated by the coefficient of determination, *R*².

### 3.2. Contact Angle of the Molten Polymers

**Figure 3:**
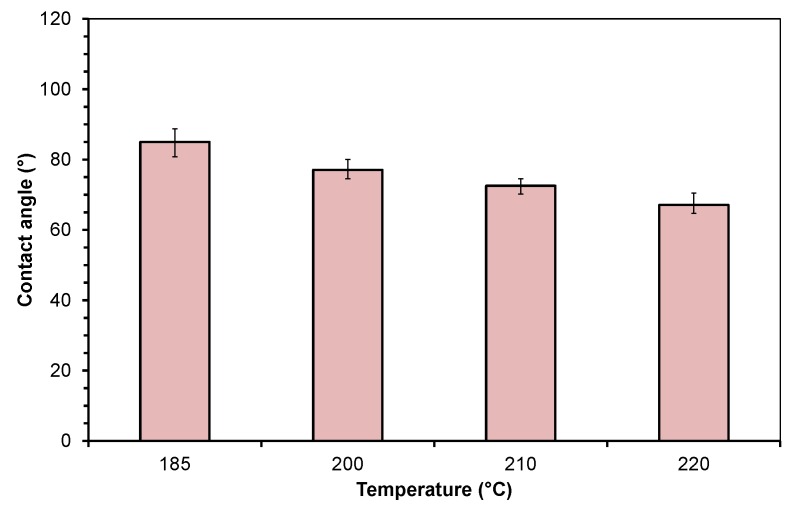
Contact angle of molten polypropylene on steel at different temperatures.

**Figure 4:**
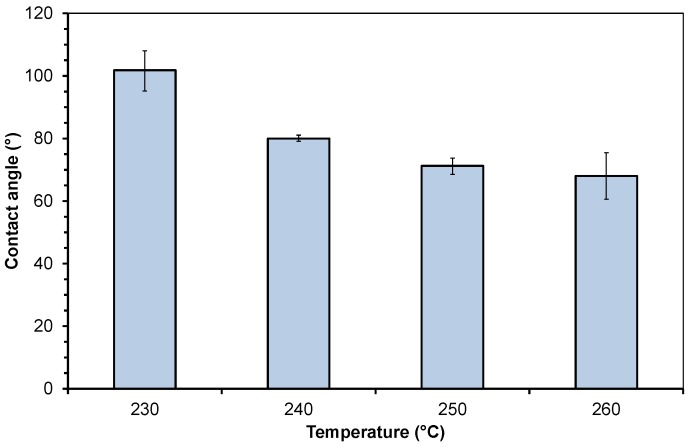
Contact angle of molten polymethylmethacrylate on steel at different temperatures.

The contact angle of the molten polymers on polished steel depends on temperature. Figure 3 and Figure 4 show the mean values of the measured contact angle data at different temperatures, including their positive and negative deviations. The contact angle values were taken at the end of the test runs to ensure stable conditions. PP and PMMA reveal a decrease in the contact angle with rising temperature. The contact angle of molten PP on polished steel is 85.0°, 77.1°, 72.6°, and 67.1° at temperatures of 185, 200, 210, and 220 °C, respectively. The low positive and negative deviations indicate the high reproducibility of the tests.

The contact angle of molten PMMA on polished steel is 101.8, 80.0, 71.3, and 68.0 °C at a temperature of 230, 240, 250, and 260 °C, respectively. At the temperatures of 230 and 260 °C, the positive and negative deviations are higher.

### 3.3. Relation Between the Contact Angle and Zero Shear Viscosity

**Figure 5:**
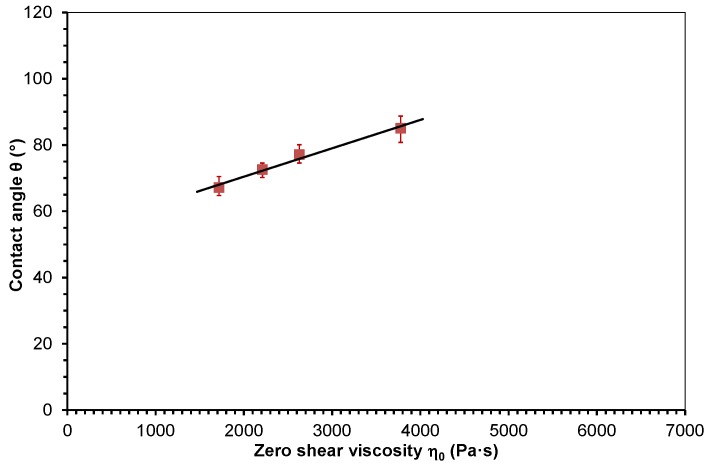
Contact angle of the molten polypropylene on steel dependent on zero shear viscosity and approximated with a linear function.

**Figure 6:**
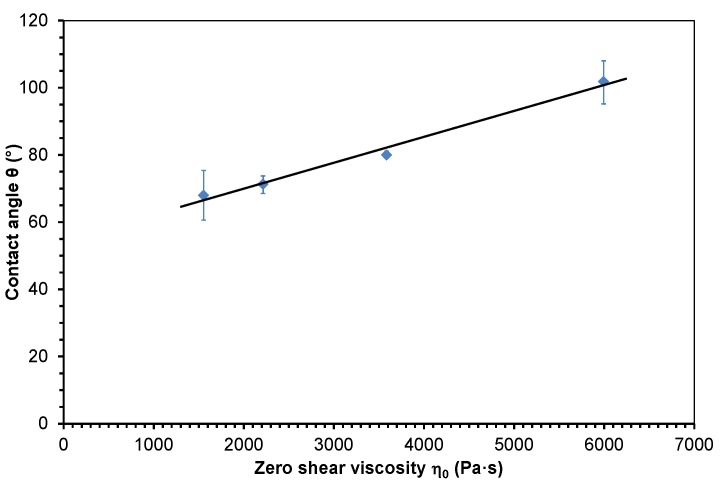
Contact angle of molten polymethylmethacrylate on steel dependent on zero shear viscosity and approximated with a linear function.

Zero shear viscosity depends on material and process parameters. The consideration that the contact angle of polymer melts on tool surfaces could be influenced in a similar manner compared to viscosity was the motivation to study the relation between the contact angle and zero shear viscosity. The mean values of the measured contact angle data were plotted in dependence of zero shear viscosity (see Table 1). The dependence of the contact angle of molten PP and PMMA on zero shear viscosity is presented in Figure 5 and Figure 6. The measured data were approximated with linear functions using the least squares method. The approximation functions and the coefficients of determination *R*² are given in Equations (7) and (8)

(7) $$\mathrm{PP}:\;\theta =0.0086\eta_{0}+53.34,\;R^{2}=0.98$$

(8) $$\mathrm{PMMA}:\;\theta =0.0077\eta_{0}+54.52,\;R^{2}=0.99$$

The values of the coefficient of determination *R*^2^ are slightly below 1, which indicates a good linear correlation between the contact angle and zero shear viscosity.

The slopes of Equations (7) and (8) are 0.0086 and 0.0077 (Pa·s)^−1^, respectively. Furthermore, the difference between the distances on the contact angle coordinate axis of Equations (7) and (8) is rather low. These values are 53.34° for PP and 54.42° for PMMA.

**Figure 7:**
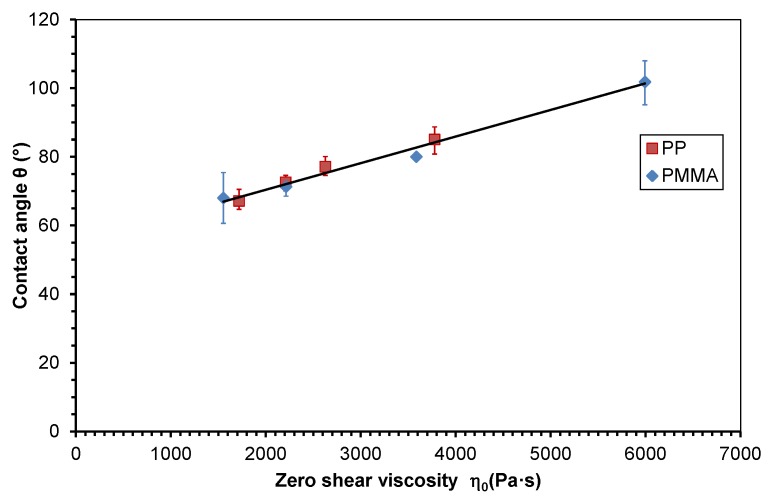
Material invariant master curve of the contact angle of molten polymers on steel dependent on zero shear viscosity and approximated with a linear function.

If the contact angle can be calculated directly from zero shear viscosity for one polymer, this equation could be the same for other polymers. The contact angle values of PP and PMMA were plotted dependent on zero shear viscosity in one diagram and approximated with one linear function to prove this hypothesis (see Figure 7)

(9) $$\theta =0.0077\eta_{0}+54.92,\;\;R^{2}=0.98$$

Although PP and PMMA are completely different polymeric materials, the relation between contact angle and zero shear viscosity follows one function, which is supported by the coefficient of determination *R*² of 0.98. Now, the contact angle of a polymer melt on polished steel can directly be calculated from zero shear viscosity of this material using Equation (9), but for a generalization of this finding, further experiments with other polymers are required.

### 3.4. Implementation of a Temperature Shift Factor for the Contact Angle

Zero shear viscosity η_0_ at different temperatures *T* can be calculated from zero shear viscosity at a reference temperature *T*_0_ employing the temperature shift concept

(10) $$\eta_{0}\left(T\right)=\eta_{0}\left(T_{0}\right)a_{T,\eta }\left(T\right)$$

where *a*_T,η_ is the temperature shift factor for shear viscosity.

The temperature shift factor of viscosity can be expressed at temperatures, *T*, well above the glass transition temperature *T*_g_ (*T* > *T*_g_ + 100 °C) using Arrhenius’ law [25]

(11) $$a_{T,\eta }\left(T\right)=\left[\frac{E_{0}}{R}\left(\frac{1}{T}-\frac{1}{T_{0}}\right)\right]$$

where *E*_0_ is the activation energy and *R* is the gas constant. The glass transition temperature is 108 °C for PMMA and 0 °C for PP, respectively.

It is assumed that the contact angle θ(*T*) at a certain temperature can be calculated from the contact angle at a reference temperature θ(*T*_0_) using a temperature shift factor *a*_T,__θ_

(12) $$\theta \left(T\right)=\theta \left(T_{0}\right)a_{T,\theta }\left(T\right)$$

Because of the linear relation between contact angle and zero shear viscosity and under consideration of Equation (9) the slope k can be expressed as

(13) $$k=\frac{\theta \left(T\right)-\theta \left(T_{0}\right)}{\eta_{0}\left(T\right)-\eta_{0}\left(T_{0}\right)}=0.0077$$

Introducing Equations (10) and (12) in Equation (13) yields

(14) $$k=\frac{\theta \left(T_{0}\right)\left[a_{T,\theta }\left(T\right)-1\right]}{\eta_{0}\left(T_{0}\right)\left[a_{T,\eta }\left(T\right)-1\right]}$$

Then, the temperature shift factor *a*_T__,θ_ for the contact angle can be expressed from Equation (14)

(15) $$a_{T,\theta }\left(T\right)=k\cdot \frac{\eta_{0}\left(T_{0}\right)}{\theta \left(T_{0}\right)}\cdot \left[a_{T,\eta }\left(T\right)-1\right]+1$$

After introducing Equation (15) in Equation (12), the contact angle θ can be calculated at different temperatures *T* from zero shear viscosity at reference temperature η_0_(*T*_0_) and from the temperature shift factor of viscosity *a*_T__,__η_

(16) $$\theta \left(T\right)=0.0077\eta_{0}\left(T_{0}\right)\left[a_{T,\eta }\left(T\right)-1\right]+\theta \left(T_{0}\right)$$

**Figure 8:**
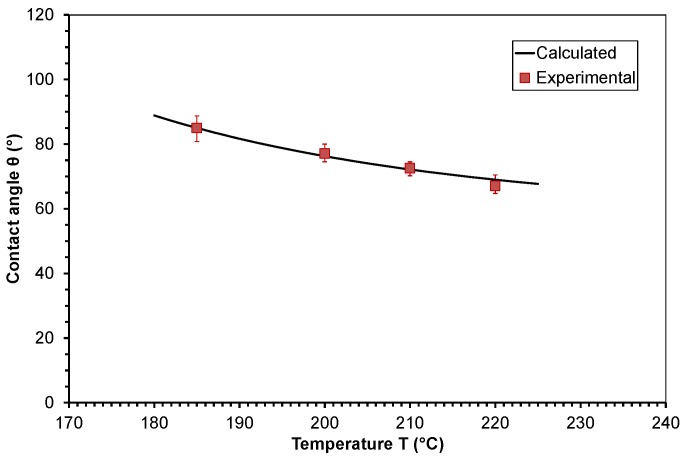
Comparison of the contact angle calculated from viscosity parameters to experimental determined values of molten polypropylene on steel dependent on temperature.

**Figure 9:**
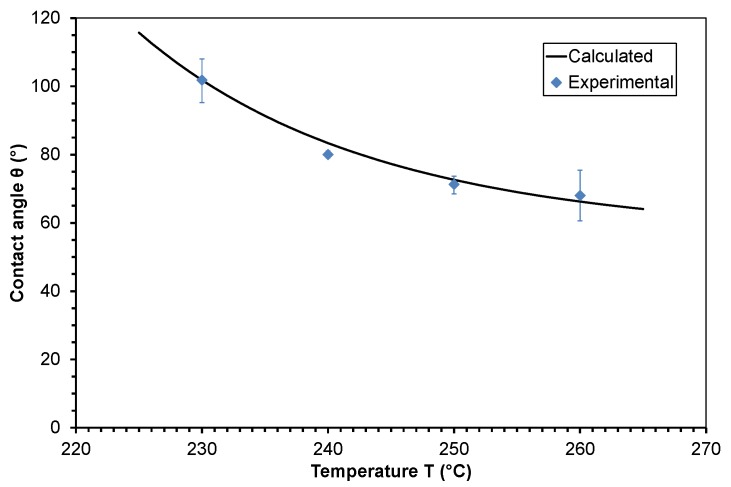
Comparison of the contact angle calculated from viscosity parameters to experimental determined values of molten polymethylmethacrylate on steel dependent on temperature.

In the next step, this model was applied to the studied polymers. The temperature shift factor of viscosity *a*_T__,η_ of the polymer melts was calculated by employing Equations (10) and (11). The zero shear viscosity values η_0_, which were determined at different temperatures (see Table 1), were shifted to the lowest temperature to obtain the activation energy, *E*_0_. A mean value for the activation energy was calculated from these data. The evaluated parameters for the calculation of the contact angle θ of PP and PMMA according to Equation (16) are given in Table 2. A comparison of the calculated contact angle from viscosity parameters to the experimental determined values is presented in the Figure 8 and Figure 9. The negligible deviations between the calculated contact angle curve and the experimentally determined values confirm the validity of the proposed model. It has to be considered that errors in the calculated values can occur below and above the studied temperature range.

## 4. Discussion

According to Young [11], the contact angle θ of a liquid on a solid is influenced by the surface energy of the solid, the surface tension of the liquid and the surface energy between the solid and the liquid. We observed a decrease in the contact angle of polymer melts on polished steel. The reason for this dependency originates in the decrease of the surface tension of liquids and polymer melts with rising temperature. Several authors reported the decrease of surface tension with rising temperature. Eötvös [26] proposed an equation which predicts a linear dependence of the surface tension of a liquid on temperature. Other authors [16,17,18,19] observed a decrease in surface tension of epoxy resins, polyesters, PE, PC, PS, PMMA, and PA melts with rising temperature. Furthermore, the decrease in the contact angle of the molten polymers on polished steel is influenced by a change of the surface energy between steel and the polymer melts with rising temperature.

We obtained a linear relation between the contact angle of the molten polymers and zero shear viscosity. This means that the contact angle increases with rising zero shear viscosity. It has to be considered that zero shear viscosity can be influenced by temperature, pressure, and the average molecular weight. The used zero shear viscosity values were obtained from the rheological measurement of shear viscosity curves at different temperatures and fit the well-known concept of temperature shift of viscosity. This temperature shift can be calculated using the Arrhenius Equation (see Equation (11)) or the Williams-Landel-Ferry Equation [27]

(17) $$loga_{T,\eta }\left(T\right)=\frac{-C_{1}\left(T-T_{0}\right)}{C_{2}+T-T_{0}}$$

which both depend exponentially on temperature. Herein, *C*_1_ and *C*_2_ are material specific parameters and *T*_0_ is the reference temperature.

In this study, we found that based on the linear correlation between the contact angle of molten polymers and zero shear viscosity the temperature shift concept can also be applied to the contact angle of dependence of zero shear viscosity also determine temperature dependence of the contact angle polymer melts on steel surfaces. The Arrhenius Equation is based on the thermally activated overcoming of energy barriers in rotational potentials of molecule segments. When considering a melt drop on a steel surface, temperature change will lead to a rearrangement of the polymer chains so that the equilibrium of forces in the three-phase point between melt, atmosphere, and solid substrate remains fulfilled. We state that the same mechanisms which determine temperature of polymer melts on steel.

It was also shown that the obtained relation between the contact angle and zero shear viscosity is a linear function, independent of the polymer used. This relation was verified for PP and PMMA. Other polymers have to be studied to enable a generalization of this relation between the contact angle and zero shear viscosity.

Furthermore, zero shear viscosity depends on the average molecular weight. The average molecular weight and the width of the molecular weight distribution influence shear viscosity curves. When polymer grades with the same distribution width but different average molecular weight are compared, the viscosity curve with the higher average molecular weight is shifted to higher zero shear viscosity values. The reason can be found in zero shear viscosity η_0_, which exhibits a dependence on the average molecular weight ${\bar{M}}_{w}$ [28]

(18) $$\eta_{0}\sim {{\bar{M}}_{w}}^{a}$$

where *a* is approximately 3.4. This means that zero shear viscosity of the same polymer increases with rising average molecular weight according to a power law. This study does not yet include grades with different average molecular weight, but there are plans to study in future work if molecular weight influences the contact angle in a similar way to temperature.

## 5. Conclusions

Currently, the contact angle of molten polymers on solid substrates can only be determined in time consuming experiments at different high temperatures, but shear viscosity curves are often more easily available for polymeric materials. We demonstrate in this paper that viscosity influences the wetting of steel by molten polymers. The contact angle of molten polypropylene and polymethylmethacrylate on polished steel was directly calculated from zero shear viscosity. The obtained linear equation is valid for both polymers and allows the assignment of a certain zero shear viscosity to a contact angle value. Furthermore, for the wetting of solid surfaces by molten polymers as it takes place in injection molding, a lower viscosity is advantageous because the contact angle decreases with falling zero shear viscosity. The zero shear viscosity depends strongly on temperature and can be assigned with a shift factor to a certain temperature. Since temperature dependence of the wettability is of great interest, we proposed a model to calculate the contact angle at different temperatures directly from the temperature shift factor of viscosity, zero shear viscosity, and the contact angle at a reference temperature. The validity of our model is confirmed by the good agreement with the experimentally determined contact angle values. As a prospect for future work, other polymers and tool materials have to be studied to enable a generalization of the relation between the contact angle and zero shear viscosity.

## Figures and Tables

**Table 1:** Bird-Carreau-Yasuda parameters of the polymers PP and PMMA at different temperatures *T* including the zero shear viscosity η_0_, the time constant λ, the Power Law index *n*, the width of the transition region *a*, and the coefficient of determination *R*².

Polymer	*T* (°C)	η_0_ (Pa·s)	λ (s)	*n* (-)	*a* (-)	*R*²
PP	185	3777.92	0.1059	0.27	0.68	1.00
PP	200	2628.46	0.0368	0.15	0.64	1.00
PP	210	2210.41	0.0472	0.23	0.61	1.00
PP	220	1719.04	0.0358	0.21	0.65	1.00
PMMA	230	5993.19	0.0574	0.35	1.62	1.00
PMMA	240	3585.80	0.0141	0.08	0.85	1.00
PMMA	250	2213.47	0.0054	0.00	0.63	1.00
PMMA	260	1553.75	0.0041	0.00	0.60	1.00

**Table 2:** Reference temperature *T*_0_, activation energy *E*_0_, zero shear viscosity at reference temperature η_0_(*T*_0_), and contact angle at reference temperature θ(*T*_0_) for the calculation of the temperature dependent contact angle from viscosity parameters of the polymers polypropylene (PP) and polymethylmethacrylate (PMMA) on steel.

Polymer	*T*_0_ (°C)	*E*_0_ (kJ·mol^−1^)	η(*T*_0_) (Pa·s)	θ(*T*_0_) (°)
PP	185	42.92	3777.92	85.0
PMMA	230	109.62	5993.19	101.8
